# Local inner ear application of dexamethasone in cochlear implant models is safe for auditory neurons and increases the neuroprotective effect of chronic electrical stimulation

**DOI:** 10.1371/journal.pone.0183820

**Published:** 2017-08-31

**Authors:** Verena Scheper, Roland Hessler, Mareike Hütten, Maciej Wilk, Claude Jolly, Thomas Lenarz, Gerrit Paasche

**Affiliations:** 1 Hannover Medical School (MHH), Department of Otolaryngology, Hannover, Germany; 2 Cluster of Excellence Hearing4all, German Research Foundation, Hannover, Germany; 3 MED-EL Innsbruck, Research & Development, Innsbruck, Österreich; COMUE Sorbonne Universites, UPMC, APHP, FRANCE

## Abstract

Dexamethasone (DEX) can reduce fibrous tissue growth as well as loss of residual hearing which may occur after cochlear implantation. Little is known about the effect of local inner ear DEX treatment on the spiral ganglion neurons (SGN), which are the target of the electrical stimulation with a cochlear implant (CI). Three different clinically relevant strategies of DEX-delivery into the inner ear were used. DEX was either eluted from the electrode carriers’ silicone, released from a reservoir by passive diffusion, or actively applied using a pump based system. The effect of the locally applied DEX on SGN density, size and function was evaluated. DEX did not affect the SGN density compared to the relevant control groups. Simultaneously applied with chronic electrical stimulation (ES), DEX increased the neuroprotective effect of ES in the basal region and the hearing threshold tended to decrease. The EABR thresholds did not correlate with the relevant SGN density. When correlating the SGN number with fibrosis, no dependency was observed. DEX concentrations as applied in these animal models are safe for inner ear delivery in terms of their effect on SGN density. Additionally, DEX tends to improve the neuroprotective effect of chronic electrical stimulation by increasing the number of surviving neurons. This is an important finding in regard to clinical applications of DEX for local treatment of the inner ear in view of cochlear implantation and other applications.

## Introduction

In the preclinical and clinical field of otology *in vitro* and *in vivo* studies as well as clinical trials are seeking for pharmacotherapies of hearing loss. Glucocorticoids, especially dexamethasone (DEX), are in the researchers and clinicians focus. Next to clinical studies investigating its effect on cisplatin-induced hearing loss [1], Mènière’s disease [2, 3], tinnitus [4], and sudden idiopathic sensorineural hearing loss [5, 6] DEX is intensively used in cochlear implant optimization research on fibrosis reduction and residual hearing preservation [7–11].

Cochlear implants (CI) are electronic inner ear prosthesis for patients suffering from severe to profound sensory neural hearing loss. Audible signals are detected by a microphone, processed into an electrical signal and transmitted transcutaneously to the implanted receiver. The signal is decoded and then delivered to the auditory nerve through an electrode array implanted into the scala tympani of the cochlea [12]. It is reported that DEX applied to cochlear implanted animals preserves residual hearing [8–11] and reduces implantation related fibrosis in the inner ear [7, 8]. Stating that DEX has an effect on residual hearing and fibrosis implies that the drug has a direct or indirect effect on inner ear cells such as the sensory cells, the cells of the lateral wall or immune cells and inflammatory mediators. It cannot be excluded that DEX additionally has—positive or negative—effects on the primary auditory neurons, the spiral ganglion neurons (SGN). These cells are necessary for the physiological hearing as well as for the efficacy of cochlear implants [13]. Correlations between functional assessments and histology in animal models suggest that proper functioning of CIs depends on the presence of a healthy and sufficiently large population of SGN that is able to transduce the encoded auditory information to the brainstem [13, 14]. In human CI users such a relation between the number of surviving SGN and CI performance was not detected [15, 16] but it is widely accepted that SGN survival and health has to be supported for a well-functioning CI-electrode-nerve interface.Therefore it is of importance that a drug, applied locally to the inner ear or entering the ear after middle ear or systemic application by diffusion, is harmless to SGN. Even though glucocorticoids are widely used and nearly half the general population aged >40 years receives at least one glucocorticoid prescription of any preparation over a 3-year period across a wide range of indications and doses [17] we still lack information about the safety of local glucocorticoid therapy on inner ear tissue [18], especially SGN. Worsøe and colleagues (2010) evaluated the effect of intratympanic betamethasone treatment on SGN loss in a rat model of experimental pneumococcal meningitis. They applied a total treatment dose of 0.27–0.54 mg betamethason-21-acetat followed by additional 0.27–0.54 mg betamethasone within 3 days. The intratympanic steroid significantly increased the number of viable neurons in the spiral ganglion [19]. In contrast, 0.2 mg/kg and 2.0 mg/kg DEX did not cause significant differences in spiral ganglion densities compared to control groups 1 month after intravenous application [11]. This correlates with the findings of Maini et al. (2009), who placed a sponge adsorbed with either 5 μl of 2% (w/v) dexamethasone phosphate or saline to the round window thirty minutes prior to implantation, and did not find changes in SGN densities one month after treatment. A change in neuronal survival became evident only 3 months after implantation when the SGN density in DEX treated ears was significantly higher than in saline treated ones [20]. The intratympanic application of DEX for treatment of Meniere’s disease is already tested in clinical trials with no concerns about DEX safety when applied outside the inner ear [21]. Local DEX application from a silicone rod implanted into the scala tympani of guinea pigs is published by Liu and colleagues. They did not evaluate the SGN number or density but the TNF-α expression of SGN [22]. The number of TNF-α -specific cells and total cells in the spiral ganglion in the first turn of controls and DEX-treated animals was counted and no statistically significant difference was observed [22]. To our knowledge there is only one report on DEX effect on SGN number with DEX applied locally into the inner ear. Stathopoulos and colleagues implanted normal hearing guinea pigs with coated CIs with an estimated DEX content of 74μg, 89μg or 100μg DEX acetate [23]. No information on the release rates of DEX out of the coating is given and no significant differences in biological effects between the DEX and the control array implanted groups were observed after 90 days of implantation [23]. Therefore the fact that they detected no effect of DEX on the number of SGN may be due to an insufficient release of the compound out of the implant to cause any biological effect.

Since currently application of DEX during CI surgery is becoming more common [24], safety data on DEX application to the inner ear are needed. We evaluated SGN number, diameter and functionality of three animal models (normal hearing, normal hearing with electrode insertion trauma and systemically deafened). Three different study protocols for DEX application were applied and for each study the release rate of DEX is given. To our knowledge this is the first report on DEX effect on SGN health with DEX applied locally into the cochlea in inner ear trauma models mimicking the situation in CI patients and applying simultaneously chronic electrical stimulation.

## Material and methods

### Animals

Normal hearing Dunkin Hartley (study I and II; Harlan-Winkelmann GmbH, Borchen, Germany) or pigmented guinea pigs (study III; Charles River WIGA GmbH, Sulzfeld, Germany) of both sexes, weighing about 250-500g, were used. Animals were maintained in accordance with current regulations (German Animal Welfare Law, ETS123, Directive 2010/63/EU). All experiments were conducted in line with these regulations and were approved by the Local Institutional Animal Care and Research Advisory Committee (IACUC) and permitted by the local authority (Lower Saxony State Office for Consumer Protection, Food Safety, and Animal Welfare Service). Three different application routes of DEX were used in three independent experimental setups. Table 1 gives an overview.

**Table 1:** Overview experimental groups.

Study	Application; hearing status	Experimental group short name	Treatment and mean release rate/h	Treatment duration [days]	Number of ears analysed	ES
I	DEX in hydrogel filled reservoir; NH	PBS	No DEX release	28	12	-
		DEX	0.35 μg DEX/hr	28	14	-
II	DEX in CI silicone; NH with insertion trauma	0% DEX	No DEX release	91	9	-
		1% DEX	0.66 ng DEX/hr	91	9	-
		10% DEX	2.04 ng DEX/hr	91	9	-
III	DEX pump; systemically deafened	AP-ES	No DEX release	27	7	-
		AP+ES	No DEX release, ES	27	6	X
		DEX-ES	25 pg DEX/hr	27	6	-
		DEX+ES	25 pg DEX/hr and ES	27	5	X

AP: artificial perilymph; CI: cochlear implant; DEX: dexamethasone; ES: chronic electrical stimulation; PBS: phosphate buffered saline; X: analysis performed; NH: normal hearing.

All surgical interventions as well as ABR measurements were conducted under general anaesthesia using ketamine and xylazine. Animals received analgesia, antibiotics and peri-operative treatment as previously described (study I: [8], study II:[7], study III: [25]).

### DEX delivery devices

**Fig 1:**
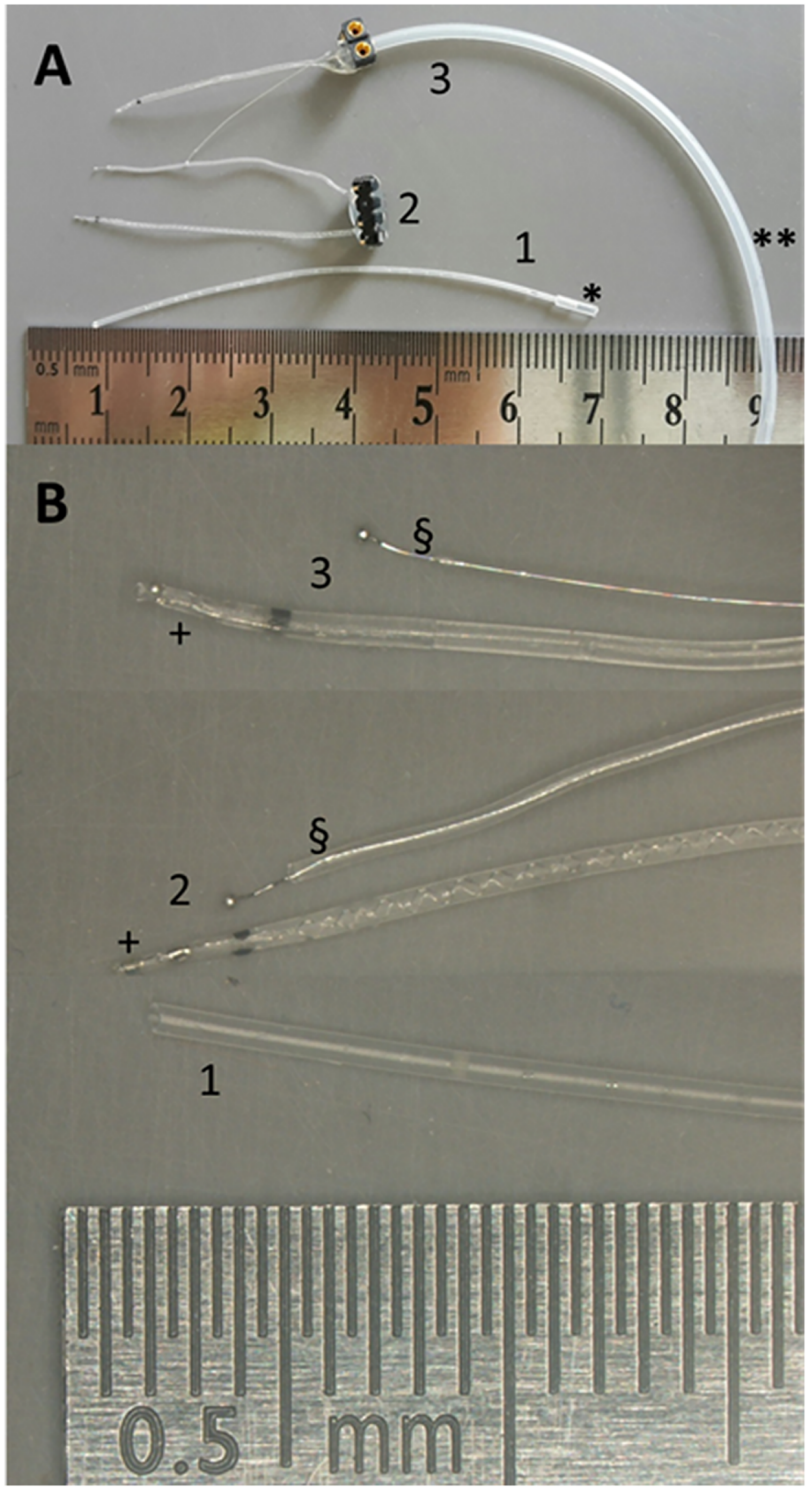
Drug delivery devices. Images of the three drug delivery systems used (A) and higher magnifications (B) are shown. The hydrogel reservoir (1) was filled via a port (*). Both, the DEX eluting electrode (2) and the electrode-micro-pump-system (3) were made of an active electrode array (+) and a reference electrode (§). The drug was administered either via diffusion from the reservoir tip (1) or from the electrode arrays silicone (2) or by active pump-based delivery through a micro-channel inside the electrode array and lead (**) connected to a pump (3; pump not shown).

**Study I, reservoir**: A hollow silicone reservoir (6 cm length, outer diameter 0.64 mm, 4.32 μL total volume, Fig 1, no.1) was filled with NCO-sP(EO-stat-PO) based dry hydrogel. Prior to implantation the reservoir was filled with either 50 μg/μL dexamethasone sodium phosphate (DEX; Spectrum chemical MFG. Corp., Gardena, CA, USA) dissolved in phosphate buffered saline (PBS) or pure PBS (for details please see Hütten et al., 2014). DEX was released from the reservoir at the tip by diffusion. The mean release rate in the first 28 days measured by ELISA was 0.35 μg/hr [8]. In the DEX-group eight animals were implanted unilaterally and 3 animals bilaterally. Resulting in n = 14 ears in total. The control group received PBS unilaterally in n = 8 animals and bilaterally in n = 2 animals, resulting in n = 12 ears for analysis.

**Study II, silicone**: The silicone of a CI electrode array (Fig 1, no. 2) was loaded with 1% or 10% DEX (DEX powder, Sigma-Aldrich) or was left unloaded (0% DEX). Over the experimental period of 91 days the mean release rates in 1%- and 10%-loaded electrodes were 0.66 ng/hr and 2.04 ng/hr respectively [7]. For each treatment group n = 9 animals were implanted unilaterally. The diameter of the electrode array was 0.6 mm at the 3 mm marker point.

**Study III, pump**: An electrode-micro-pump-system was used to deliver a 100 ng/mL DEX-solution (Fortecortin^®^ Inject, Merck, Germany) dissolved in artificial perilymph (AP) into the inner ear (Fig 1, no. 3). Via a silicone tube the active electrode system was attached to an osmotic pump with a release rate of 0.25 μL per hour (model 2004, ALZET, Durect Corp., Cupertino, California, USA), resulting in delivery of 25 pg DEX per hr. The control group was implanted with an electrode-micro-pump-system delivering AP only. All animals of study III were implanted unilaterally. The outer diameter of the electrode array was 0.6 mm.

### Hearing status, surgical procedure and ABR

Three different animal models were used to mimic differences in the initial hearing status. The normal hearing of all animals was confirmed by measuring acoustically evoked auditory brainstem responses (AABR).

Subsequently, animals of **study I and II** were implanted with their respective DEX-delivery device.

Animals of **study I**, receiving the hydrogel reservoir, displayed the situation of implanting a normal hearing individual.

The DEX-eluting electrode of **study II** was inserted three times and withdrawn 2 times before leaving it in place. Those animals are a model for electrode insertion trauma induced by the implantation of a CI [7].

Animals of **study III** were systemically deafened using kanamycine i.m. and ethacrynic acid i.v. to induce profound hearing loss. Deafening, defined as a threshold shift of 60 dB or more, was confirmed after 21 days by measuring AABR and animals were implanted using the respective electrode-micro-pump-system.

Before surgery DEX solutions, PBS and AP were prepared freshly and filled into the reservoirs, pumps and connected tubes. Using a postauricular approach the middle ear cavity was opened and the cochlea was visualised. For implantation of the hydrogel reservoir a cochleostomy was drilled ventral to the round window in the basal turn of the cochlea (diamond burr, 600 μm in diameter) and the reservoir was inserted 3 mm deep into the scala tympani. To implant the DEX-eluting electrode array (study II) and the electrode delivering DEX-solution (study III) the round window membrane was incised and the relevant DEX-delivery device was inserted approximately 3 mm into the scala tympani. In study III where a chronic electrical stimulation was envisaged the reference electrode was placed intratympanically on the bony wall of the bulla.

In all groups the implant was fixed and the fenestration of the middle ear closed using dental cement (Durelon, ESPE Dental AG, Seefeld, Germany). Thereafter, the protruding part of the reservoir was rolled-up and secured subcutaneously, the pump was implanted subcutaneously between the scapulae and the wound was sutured in two layers.

Beginning on day 24 (3 days after implantation), animals of the AP+ES and DEX+ES groups (Table 1) of study III received continuous pulsatile ES for 24 days. Biphasic charge balanced pulses with 100 μsec per phase, at 250 Hz were presented 8 dB above the electrical response threshold using a battery-powered, wearable stimulator [26, 27]. The hearing threshold was recorded by electrically evoked auditory brainstem response (EABR) measurements directly after electrode implantation (day 21 after deafening) and weekly for four weeks [25].

### Exploitation of specimens and histological preparation

On day 28 (**study I, reservoir**), 91 (**study II, silicone**) or 27 (**study III, pump**) after starting the DEX application guinea pigs were anesthetised, final ABR measurements were performed and animals were sacrificed by PBS perfusion. Tissue was fixed using modified Wittmaack solution (study I and II) or glutardialdehyde (GDA; study III). The drug delivery devices were cut next to their entrance into the bulla, the temporal bones were harvested and the otic capsule was trimmed. Drug delivery devices of study I and II were kept in place while the electrode arrays of study III were explanted. The *fenestra ovalis* and the apex were pierced with a lancet and the specimens were rinsed and fixed in modified Wittmaack fixing solution **(study I and II)** or GDA (**study III**) for up to 24 hours.

To allow histology of **study I and II specimens** with the DEX-delivery devices in situ, plastic embedding was performed: they were washed for 10 hours with a 4% solution of lithium sulphate and dehydrated (2 hours per concentration) in ascending ethanol concentrations (50% v/v, 70% v/v, 90% v/v and 100%) or optionally overnight in 70% v/v ethanol. Subsequently, cochleae were dried at room temperature for at least 12 hours and embedded in 5 parts epoxy resin and 2 parts hardening agent (SpeciFix-40 Kit, Struers GmbH, Ballerup, Denmark). Resin was adjusted by the addition of titanium oxide (Merck KGaA, Darmstadt, Germany) for whitening and reduction of transparency and the samples were left to harden at room temperature. All plastic embedded specimens were grinded with a grinding machine (PowerPro 4000, Bühler, Lake Bluff, Illinois, USA) and abrasive paper. For every layer of the cochlea, 20 μm were abraded and the section was stained for two minutes with each eosine and toluidine. The freshly stained surface of the specimen was photographically documented at 30-, 150- and 200-times magnification using a Keyence system (VHX-600 DSO, Osaka, Japan).

In **study III specimens** were rinsed with tri-sodiumcitrate and subsequently decalcified for about 2 weeks in a trisodium citrate—formic acid mixture; the solution was changed every day. Finally, cochleae were dehydrated through a graded series of alcohols (70–100% ethanol), embedded in paraffin, and serially sectioned at 5 μm in a midmodiolar plane. Each section was mounted on a glass slide and stained with hematoxylin and eosin.

### Spiral ganglion neuron assessment

**Fig 2:**
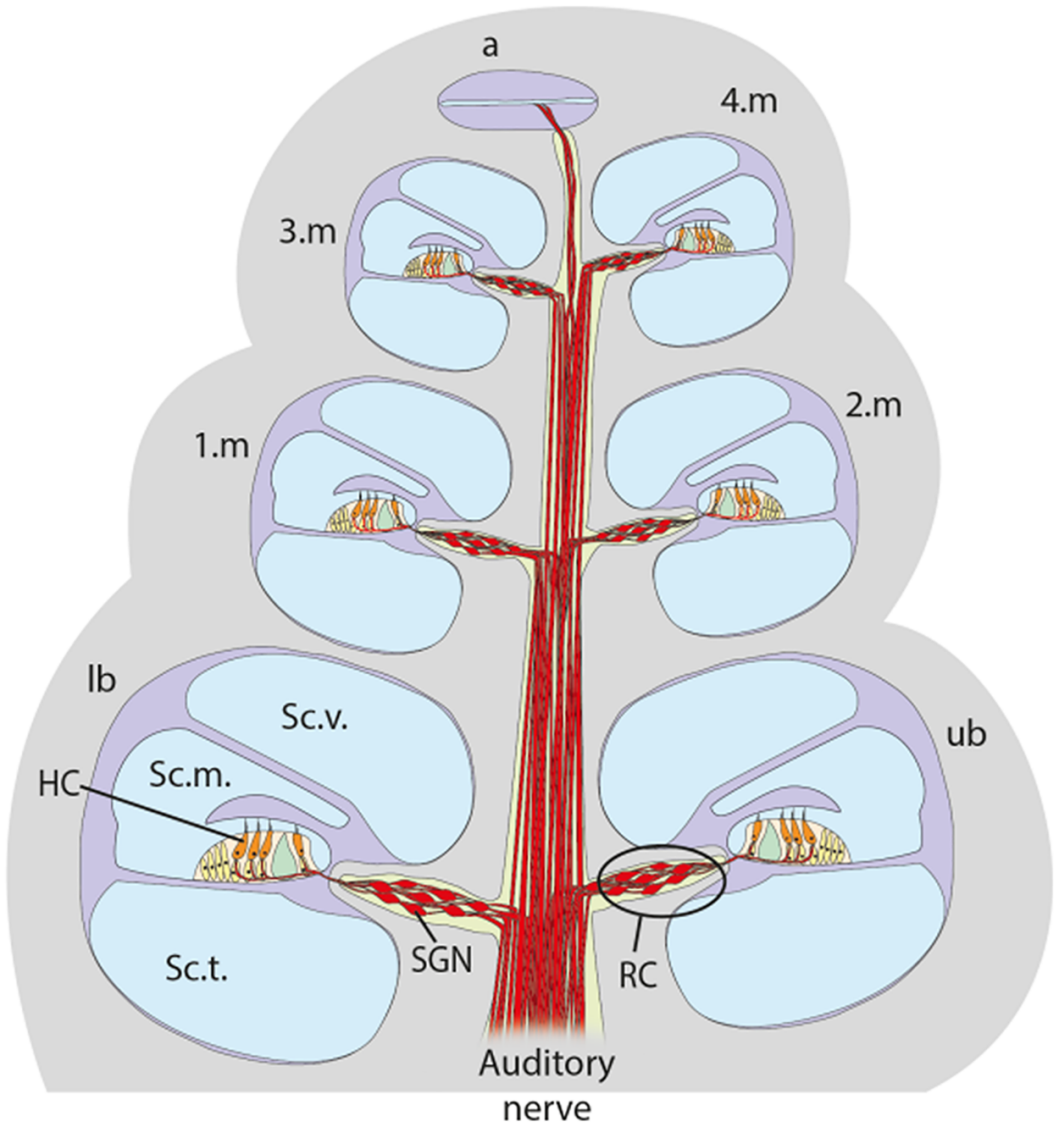
Drawing of a normal hearing guinea pig cochlea in a midmodiolar plain. The cochlea consists of three separate membranous compartments (Scala tympani, Sc.t.; Scala media, Sc. m.; Scala vestibuli (Sc.v.) curled around the bony axis, the modiolus, which contains the Rosenthal’s canal (RC) including the primary auditory neurons, the spiral ganglion neurons (SGN). Each midmodiolar cross section of the cochlea contains 6 to seven cross sections of the Rosenthal’s canal (lb to a), and the SGN were counted in each of these cross sections. SGN diameters were measured in three ipsilateral Rosenthal’s canal cross sections (lb, 1.m and 3.m turns).

For SGN analysis in all three studies all cochlea turns were identified microscopically. The turns were named lower basal turn (lb), upper basal turn (ub), first middle turn (1m), second middle turn (2m), third middle turn (3m), fourth middle turn (4m), and apical turn (a) (Fig 2). Due to preparation methods, the 4m and a turns could not always be analysed separately. Therefore, the SGN assessments of these areas, if available, were added for analysis. The first midmodiolar section or plane, respectively, was selected randomly.

In **study III** every fifth following section was chosen for analysis to ensure a separation of 25 μm between the sections.

In **study I and II** planes of minimum 25μm distance were evaluated to assure that no cell was counted twice. In total, five sections/planes per cochlea were analysed [28].

SGN were displayed at a magnification of 200x. In each of the 7 or 6 different cross sections of the Rosenthal’s canal the number of surviving SGN in correlation to the measured respective cross-sectional area of the Rosenthal’s canal provides the SGN density, which can be expressed as cell number/10.000 μm^2^.

**Study I and II**: SGN counting was based on Ceschi (2013) who compared the soma size of SGN from 6 normal hearing guinea pigs fixed with Wittmaack solution and embedded in epoxy as used in the current study with SGN fixed with paraformaldehyde and encased in paraffin [29]. In this study, based on the different shrinking factor of the tissue due to the differences in fixation and embedding, a minimum diameter of viable SGN somata from normal hearing animals embedded in epoxy of at least 8.4 μm was determined according to the 12 μm as otherwise given in literature [30]. Accordingly, we set 8.4 μm as minimum diameter for viable SGN and counted only those neurons in our SGN which had a 8.4 μm or larger soma diameter.

**Study III**: specimens were embedded in paraffin, all SGN per Rosenthal’s canal with a minimum perikaryal diameter of 12 μm were counted and included for analysis as described in literature [25, 30–32].

In all three studies perikaryal diameter of 5 randomly selected SGN were assessed in the lb, 1m and 3m Rosenthal’s canal cross sections of the 5 sections already used for SGN count.

### Fibrosis assessment

The effect of locally applied DEX on fibrosis in studies I (reservoir) and II (silicone) are published (study I: [8]; study II: [7]). Study III (pump) data on the effect of DEX on fibrosis are published here.

For assessment of fibrosis inside the *Scala tympani*, **study I and III** used a subjective ranking to describe the fibrosis in the basal turn of the *Scala tympani*. In study I connective tissue was assessed in the first and the last midmodiolar plane section as well as the one in the center. In study III for each of the five layer/slides, analysed for SGN assessment, a ranking system from score 0: no connective tissue; score 1: less than one quarter of the scala tympani is filled with connective tissue; score 2: less than half but more than one quarter of the scala is occupied by tissue and/or the whole surface of the implants crosscut is covered; to score 3: more than half of the scala tympani is filled with connective tissue; was used [8].

In **study II** three zones of the basal *Scala tympani* were analysed to detect the connective tissue growth: the area of the round window niche, the basal region behind the niche and the first turn behind the modiolus. To calculate the percentage of fibrous tissue growth, the area of the scala tympani was accessed by tracking the inner outline of the scala tympani using Keyence software (VHX Communication Software for VHX-2000). From this outlined section, the part occupied by the electrode was subtracted to obtain the free area of the scala tympani which could potentially be filled with connective tissue. This area was normalized to 100%. The area filled with new tissue was identified using the same system and software as above. The percentage of *Scala tympani* filled with connective tissue was calculated in relation to the selected normalized area (100%) [7].

### Statistical analysis

Statistical analysis was performed using GraphPad Prism^®^ 5 (GraphPad Software Inc., La Jolla, California, USA) and normality test was performed using D’Agostino & Pearson omnibus normality test, where applicable. SGN number in all three studies did not follow Gaussian distribution. Therefore in **study I**, containing two independent samples of different sizes, a Mann Whitney test was used, and in **studies II and III**, with more than two groups, the Kruskal-Wallis test was used to determine if one group stochastically dominates one of the others. Subsequently Dunn’s post-hoc test was performed to analyse the differences in SGN number between the experimental groups.

Soma diameter in **study I** groups passed normality test and an unpaired t-test was performed to analyse group differences. In **studies II and III** SGN soma diameter did not follow Gaussian distribution, Kruskal-Wallis tests and Dunn’s post-hoc tests were performed.

Median SGN numbers and soma diameter are given. Statistical comparison between the different studies was not performed due to the variability in study set ups.

EABR thresholds and amount of fibrosis in **study III** did not allow for normality testing due to the reduced data set. For EABR thresholds Wilcoxon signed rank test was performed to detect differences between medians. To determine treatment related differences in fibrosis medians the Kruskal-Wallis test was used.

Significance is defined as p<0.05 (*), p<0.01 (**), and p<0.001 (***).

The degree of correlation between SGN number or diameter and EABR threshold or between SGN number and fibrosis was assessed using Spearman correlation coefficients.

## Results

### SGN number

**Fig 3:**
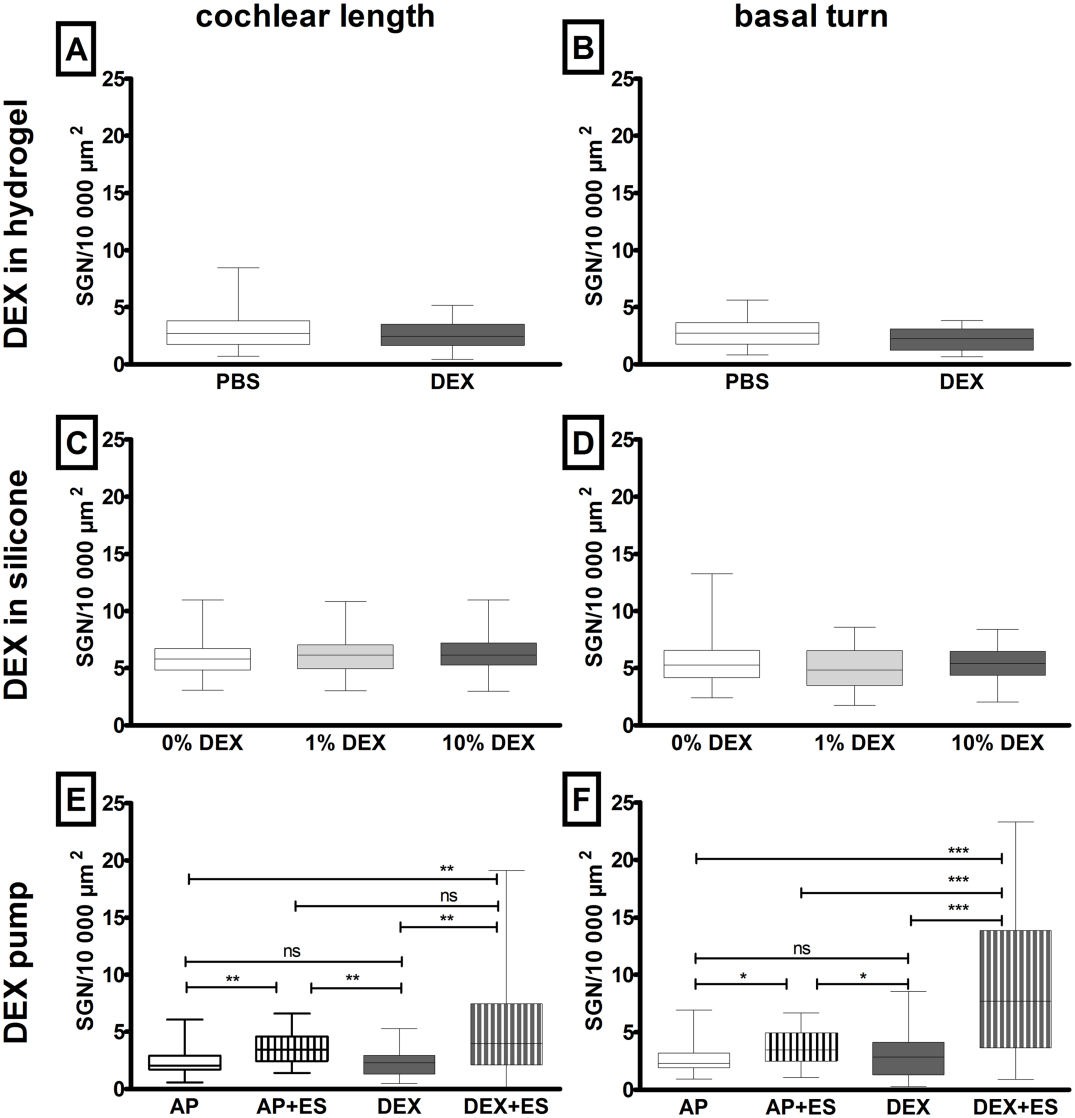
Median number of SGN. The median number of SGN per 10.000 μm^2^ in guinea pigs implanted with a hydrogel reservoir (A, B; study I), electrode array (C, D; study II) or electrode-micro-pump-system (E, F; study III) for the entire cochlear length (left graphs) and basal turn (right graphs) are plotted in box and whiskers (min. to max.). DEX did not affect the number of surviving SGN. Applied simultaneously with ES DEX increased the SGN protective effect of ES. * = p<0.05, ** = p<0.01, *** = p<0.001, ns = not significant.

**Study I, reservoir**: No differences in median SGN number/10.000 μm^2^ of Rosenthal’s canal area were detected from the lower basal turn up to the apical turn in study I (DEX: 2.45, PBS: 2.73, p = 0.15; Fig 3A). Additionally, comparing the median SGN number/10.000 μm^2^ of Rosenthal’s canal area of the basal turns, i.e. the lower and upper basal turns, between DEX (2.27 cells/10.000 μm^2^) and PBS treated ears (2.74 cells/10.000 μm^2^; Mann Whitney test, p = 0.14), no differences were observed (Fig 3A).

**Study II, silicone**: Likewise study I, the SGN numbers in study II did not vary significantly between the experimental groups when comparing the total number over the full cochlea length or numbers in the basal region (Kruskal-Wallis test; entire cochlea: p = 0.23; basal turns: p = 0.18). The implantation of an electrode array with 0% DEX resulted in a median SGN number of 5.78 cells/10.000 μm^2^ in total and 5.27 cells/10.000 μm^2^ in the basal region. 1% DEX and 10% DEX treatment caused median SGN numbers of 6.14 (total) and 4.82 (basal) and 6.13 (total) and 5.41 (basal) cells/10.000 μm^2^, respectively (Fig 3C and 3D).

**Study III, pump**: Comparing the number of SGN per 10.000 μm^2^ in ears implanted with an electrode-micro-pump-system, significant differences between treatment groups were observed when focused on the total cochlear length and the basal region (Kruskal-Wallis test, p<0.001). When comparing the median SGN number of the total cochlear length, the control group receiving AP (2.04 SGN/10.000 μm^2^) had a significantly reduced SGN number in comparison to electrically stimulated ears and ears receiving electrical stimulation and DEX (AP+ES: 3.44 SGN/10.000 μm^2^, p<0.01; DEX+ES: 3.98 SGN/10.000 μm^2^, p<0.01), but no differences were observed compared to DEX—only treated individuals (2.28 SGN/10.000 μm^2^). Additionally, the total median SGN number of AP+ES treated ears was significantly increased in comparison to DEX (p<0.01) but not to DEX+ES. The SGN number of the DEX+ES group was with p<0.01 increased compared to DEX alone (Fig 3E).

Focusing on the basal cochlear region electrical stimulation (AP+ES group, 3.47 cells per 10.000 μm^2^) increased the number of SGN significantly (p<0.05) compared to AP-only (2.32 SGN/10.000 μm^2^) and DEX-only (2.87 SGN/10.000 μm^2^) treated ones. No differences in SGN density between DEX- and AP-treated ears were observed. The number of SGN in the basal region simultaneously being treated with DEX and electrical stimulation (DEX+ES: 7.72 SGN/10.000 μm^2^) was significantly increased compared to neurons of all other experimental groups (p<0.001; Fig 3F).

### SGN soma diameter

#### A: Cochlear length

**Fig 4:**
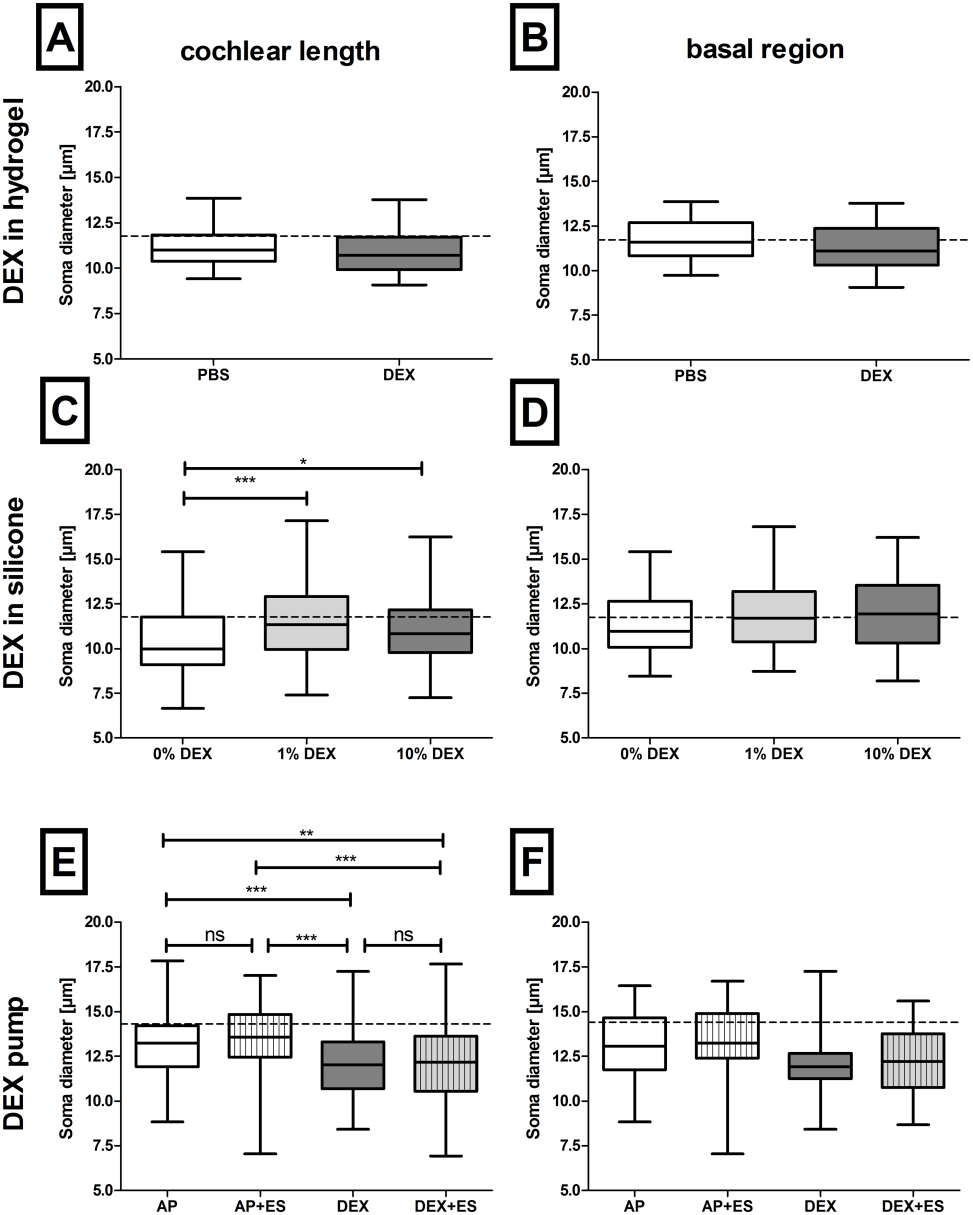
Soma diameter in DEX treated animals compared to the relevant control group. DEX treatment using a reservoir (study I, A, B) did not affect the SGN soma diameter compared to PBS treated control ears. DEX elution out of the implant in two concentrations (study II) increased the soma diameter compared to the relevant control group over the full cochlear length (C) but not in the basal region (D). Using a pump-delivery-system (study III) DEX decreased the soma diameter compared to AP- and AP+ES-treated ones in the entire cochlea (E) but not in the basal region (F). Dotted lines: mean SGN soma diameter of normal hearing animals in epoxy resin embedded specimens for the full cochlear length (11.77μm; A, C) and the basal region (11.72μm; B, D) and in paraffin embedded for the full cochlear length (14.31μm; E) and the basal region (14.4 μm; F). Box and whiskers (min to max), * = p<0.05, ** = p<0.01, *** = p<0.001, ns = not significant.

**Study I, reservoir**: 0.35 μg DEX per hour diffused out of a hydrogel reservoir into the scala tympani for 28 days. Here, no difference in group medians was observed for the entire cochlear length, that is, the basal, middle and apical parts of the cochlea (Gaussian distribution, unpaired t-test, p = 0.24) with a median soma diameter of 10.99 μm in PBS and 10.71 μm in DEX treated ears (Fig 4A).

**Study II, silicone**: Both DEX concentrations eluted out of the electrode’s silicone into the inner ear increased the SGN diameter compared to the negative control (Fig 4B; no Gaussian distribution, Kruskal-Wallis test: p<0.001, followed by Dunn’s Multiple Comparison test). Here the median SGN diameter of control ears (0% DEX) was 9.98 μm and differed significantly from 1% DEX treated ears (11.34 μm, p<0.001) and 10% DEX treated ones (10.83 μm, p<0.05).

The mean SGN soma diameter of normal hearing animals for the total cochlear length (11.77 μm) evaluated in a previous study (28) using the same histological method as in study I and II is indicated in Fig 4A and 4C to illustrate the SGN diameter of those studies related to normal. In study I soma diameter of DEX and PBS treated animals differed in the same range from normal hearing ones. In study II the soma diameter of DEX treated ears was close to normal and those of DEX-untreated controls were decreased compared to normal.

**Study III, pump**: In contrast to study I and II—where soma diameter of plastic embedded neurons were measured—in study III paraffin-embedded specimens were analysed for SGN soma diameter. The soma diameter of the full cochlear length of the four experimental groups in this study did not follow Gaussian distribution, Kruskal-Wallis test proved a significant variance between groups (p<0.001) and Dunn’s Multiple Comparison test was performed. Both drug treated groups, DEX with and without ES, showed significantly decreased median SGN soma diameter compared to the AP treated controls (DEX: p<0.001; DEX+ES: p<0.01). Chronic electrical stimulation did not influence the soma diameter (DEX (12.02 μm) vs DEX+ES (12.18 μm): ns; AP (13.23 μm) vs AP+ES (13.58 μm): ns; Fig 4E)).

#### B: Basal region

The soma diameter of SGN of the basal turns in **study I and II** passed the normality test and did not differ between groups with 11.60μm (PBS) and 11.10μm (DEX) median diameter in study I (Mann Whitney test; Fig 4B) and 10.96μm (0% DEX), 11.70μm (1% DEX) and 11.93μm (10% DEX) median diameter in study II (Bonferroni’s Multiple Comparison test; Fig 4D).

In paraffin embedded specimens as analysed for soma diameter of the basal regions in **study III**, no Gaussian distribution was observed. Kruskal-Wallis test calculated a p value of 0.02 and Dunn’s Multiple Comparison test revealed no significant differences between groups (Fig 4F).

### EABR

**Fig 5:**
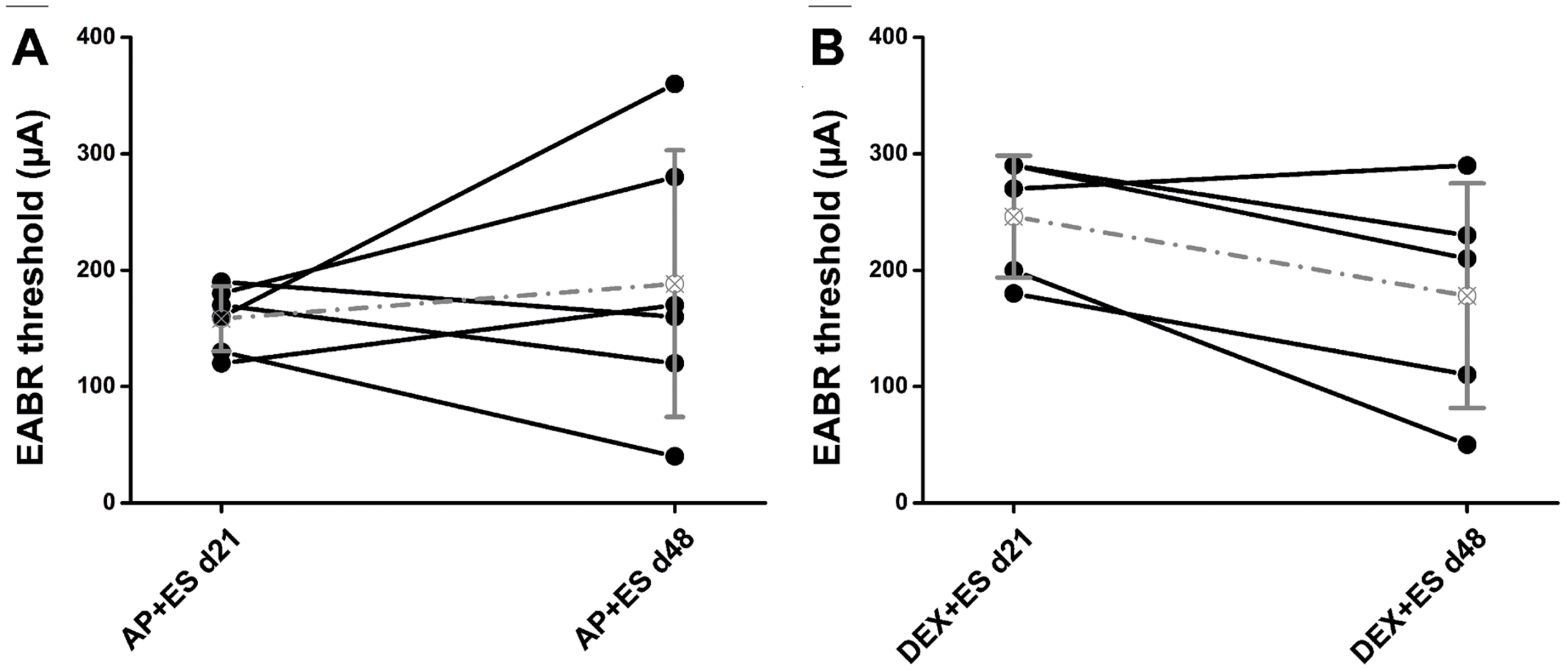
Electrical hearing thresholds. EABR thresholds in AP+ES treated animals (A) increased in 3 and decreased in 3 individuals. DEX+ES therapy caused decreased thresholds in 4 out of 5 animals. In both groups the range of thresholds was increased after stimulation. Individual thresholds are marked with black lines whereas the mean ± SD hearing thresholds are grey dotted lines.

In **study III** DEX delivery was combined with chronic ES to investigate a potential effect of both simultaneously applied interventions, mimicking the clinical situation, on SGN number and functionality. The median EABR thresholds of DEX+ES treated individuals decreased from 270 μA (mean ± SD: 246±52 μA) at the day of implantation to 210 μA (mean ± SD: 178±96 μA) after 4 weeks of therapy. Animals receiving ES only (AP+ES group) showed a median EABR threshold at the day of implantation of 165 μA (mean ± SD: 158±27 μA) which did not change over time (day 48 median: 165 μA, mean ± SD: to 188±114 μA) (Fig 5). Even though all experiments and EABR measurements were performed by one person, all animals were randomly assigned to one of the experimental groups and experiments of the two groups were performed in random order the initial EABR thresholds of both groups differed significantly (Mann Whitney test, p<0.05).

In both groups no significant differences in EABR threshold changes from day of therapy start to the final measurement were observed (Fig 5). In both groups a change from a relatively small SD (27 μA and 52 μA) to a broader one (114 μA and 96 μA) is observed. This is due to an increased EABR threshold variability in both groups after 4 weeks of treatment (Fig 5).

### Correlation EABR thresholds versus SGN number and diameter

**Fig 6:**
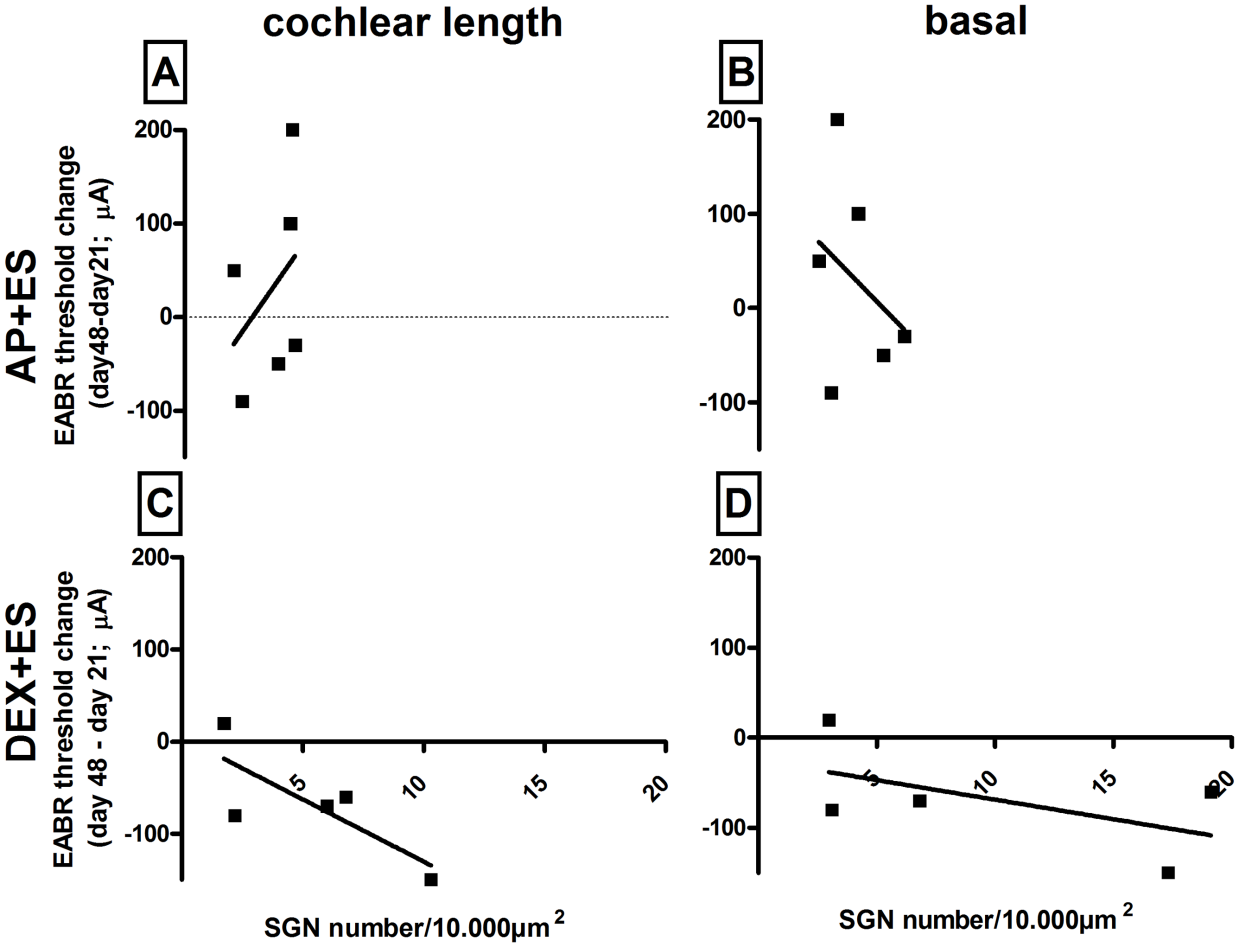
Correlation of hearing threshold change over time and SGN number. Neither the SGN number of the entire cochlea (A,C) nor of the basal region (B,D) correlated with the relevant EABR threshold shift in the AP+ES group (A,B) and the DEX+ES group (C,D).

When correlating the SGN number of the entire cochlear length (Fig 6A and 6C) or the basal region (Fig 6B and 6D) and the respective EABR threshold, no dependencies were observed for both electrically stimulated experimental groups in **study III** (Spearman correlation: AP+ES total: r = 0.20, p = 0.71; AP+ES basal: r = 0.54, p = 0.29; DEX+ES total: r = -0.70, p = 0.23; DEX+ES basal: r = -0.40, p = 0.51).

**Fig 7:**
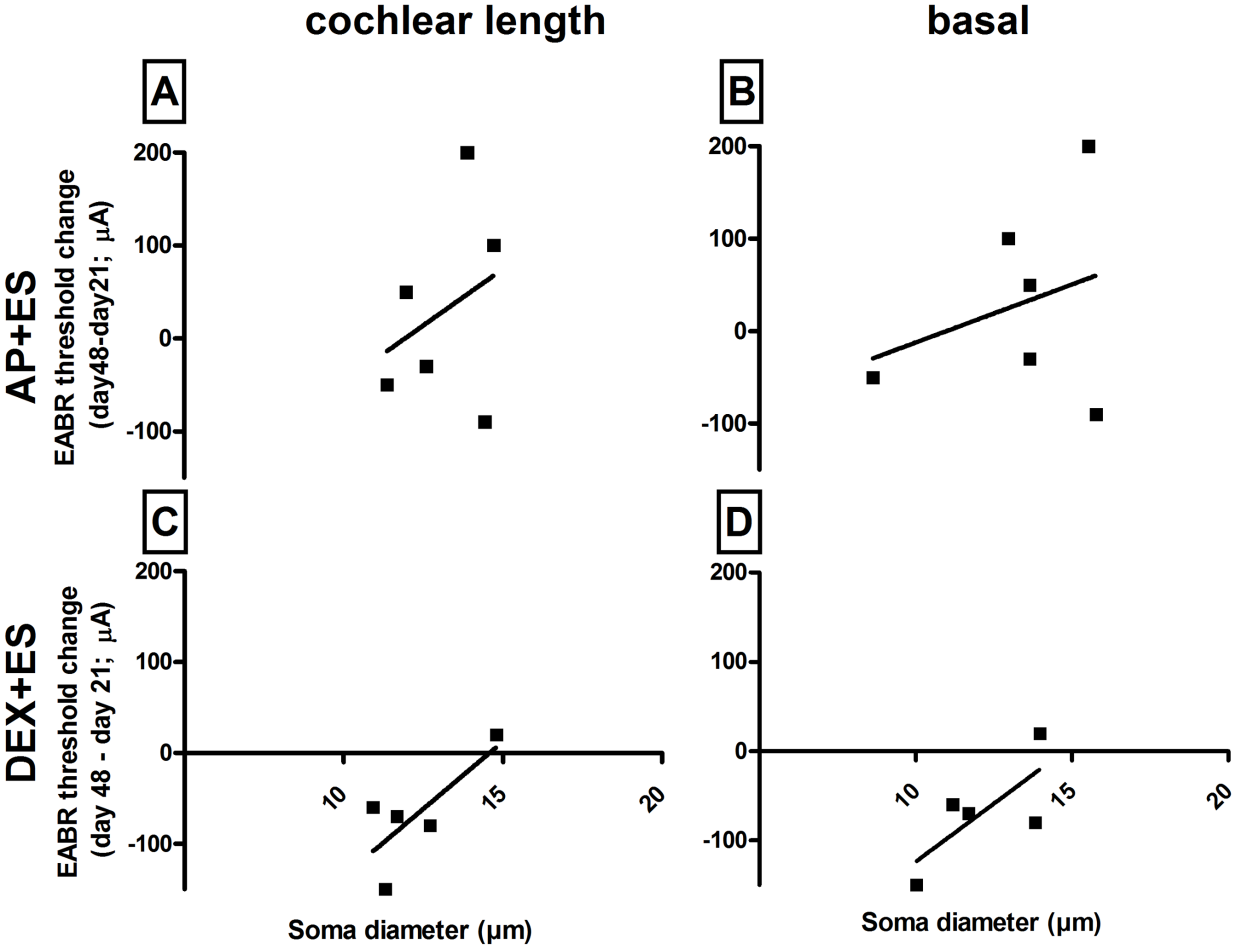
Correlation of hearing threshold change over time and soma diameter. Neither the SGN soma diameter of the entire cochlea (A,C) nor of the basal region (B,D) correlated with the relevant EABR threshold shift in the AP+ES group (A,B) and the DEX+ES group (C,D).

To elucidate a possible correlation between soma diameter and EABR threshold change over time we performed Spearman correlation test (Fig 7A–7D). No dependencies of neuron size and their functional responsiveness was determined (AP+ES total: r = 0.25, p = 0.65; AP+ES basal: r = -0.05, p = 0.91; DEX+ES total: r = 0.30, p = 0.68; DEX+ES basal: r = -0.60, p = 0.35).

### Fibrosis

**Fig 8:**
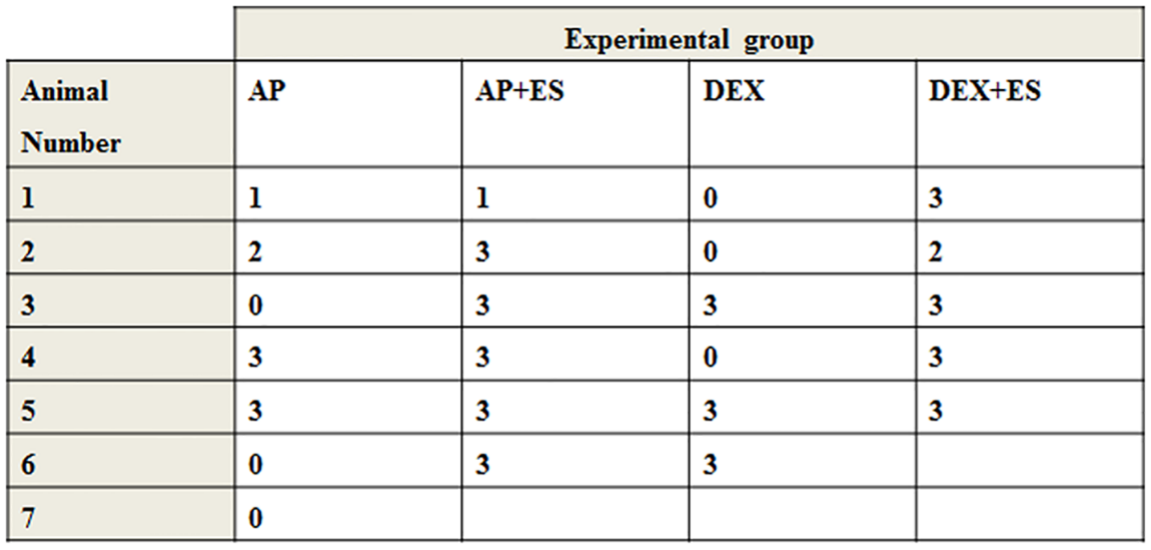
Fibrosis scores for study III. No differences in fibrous tissue growth between the experimental groups were observed.

The fibrosis decreasing effects of DEX released from a reservoir (study I) or silicone (study II) were previously published [7, 8]. DEX solution delivered by an osmotic pump—electrode-system (study III, fibrosis data for the first time published here) did not change the amount of fibrosis in implanted cochleae compared to controls nor did ES (Fig 8).

### Correlation of fibrosis and SGN number

**Fig 9:**
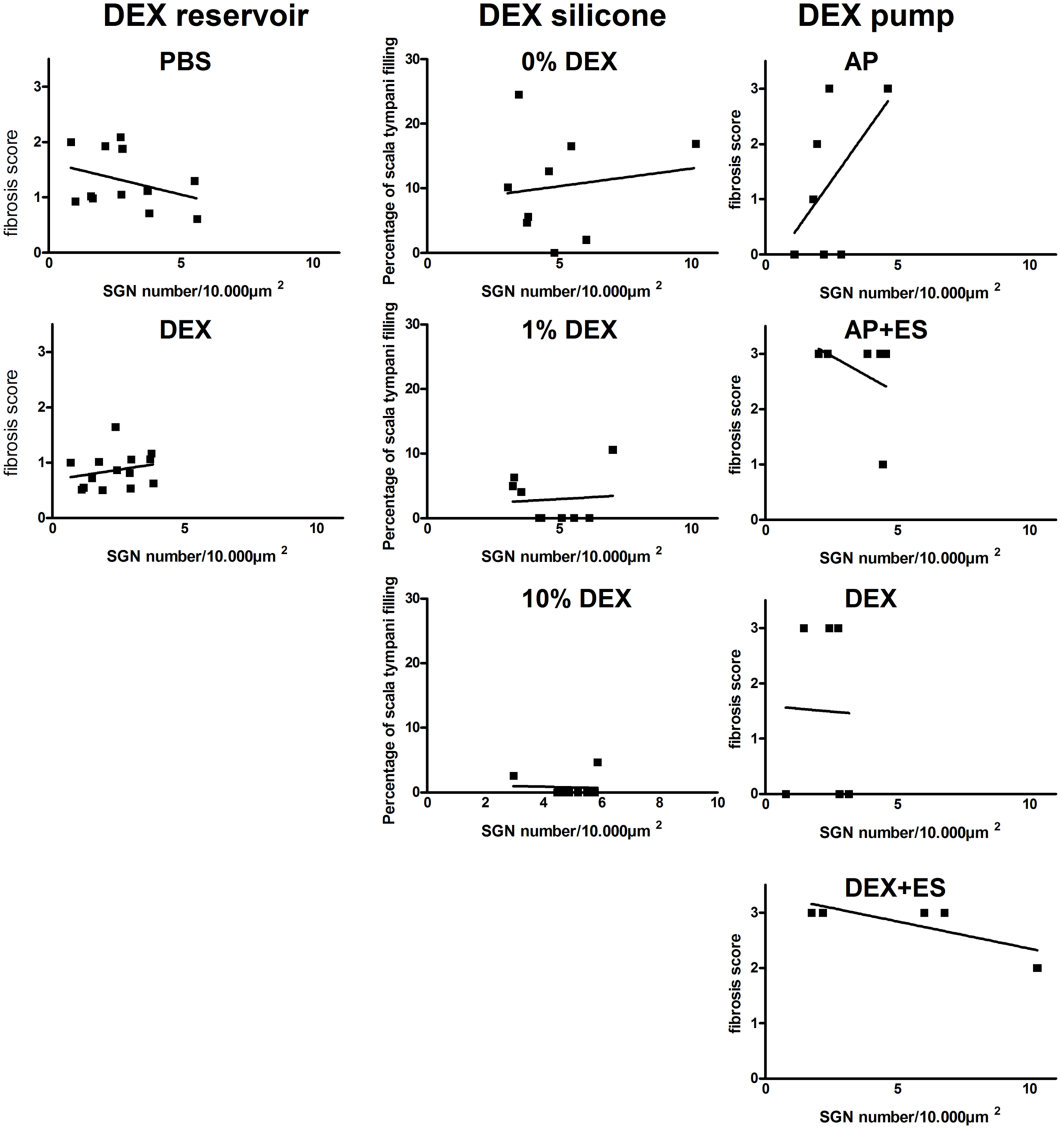
Correlation assessment of SGN number and amount of fibrosis. The SGN density (number of neurons in 10.000 μm^2^) was correlated to the relevant fibrosis score (left and right column) and the percentage of the scala tympani area filled with fibrosis (middle column). No correlations were detected.

Since it is not known if postoperative cochlear fibrosis has an effect on SGN survival (e.g. by factor release or by disturbance of nutrition flow onto the SGN) we correlated the SGN number/10.000 μm^2^ of the basal region with the relevant basal fibrosis. No correlation between SGN number and fibrosis was detected for the whole cochlear length (data not shown) or for the basal region (Fig 9).

## Discussion

### Effect of DEX on SGN

DEX is a synthetic glucocorticoid. Similar to natural glucocorticoids, DEX has a mainly inhibitory effect by modulating the protein synthesis at the transcription level or by influencing the ion transport or interference with protein phosphorylation [33]. DEX acts mainly via a cytosolic glucocorticoid receptor which is expressed in most inner ear cell types, including SGN [34, 35]. Because local DEX application to the inner ear in conjunction with cochlear implantation is becoming more and more common due to promising results related to hearing preservation [8, 36–38] or reduction of tissue formation after cochlear implantation [7, 8, 37, 39], its safety on SGN has to be verified.

#### SGN number

Previously, safety of 25, 50 and 100 ng/ml DEX on cultivated dissected SGN was reported [40]. In the present *in vivo* studies, using three different DEX application methods, concentrations and animal models, again, DEX did not affect the SGN density. Therefore we can state that DEX is not toxic to inner ear neurons, and local DEX application, as used in these studies, is safe for cochlear implanted ears in guinea pigs. This finding is in line with the results published by Stathopoulos and colleagues who implanted guinea pig inner ears using 100 μg, 89 μg and 74 μg DEX containing electrode arrays and found no difference in SGN number between treatment groups and controls [23]. But one has to keep in mind that in their study DEX did not cause any significant biological effect. In contrast, the three DEX concentrations used in the studies presented here, did affect fibrosis and electrical responsiveness of SGN. Other groups did not apply DEX directly into the inner ear but into the middle ear and evaluated its safety on inner ear structures. Piu and colleagues did not detect a DEX related change in hearing thresholds, cytocochleograms or SGN appearance in normal hearing guinea pigs receiving a single shot of Oto-104 intratympanically [41]. Additionally, Maini and colleagues did not detect an effect of DEX on SGN density 4 weeks after applying a 2% DEX phosphate containing sponge on the round window membrane. In contrast, they observed a slight SGN density increase mediated by DEX application detectable 3 months after treatment [20]. Even though we constantly applied DEX over a period of 90 days after cochlear implantation (study II) we did not find such an effect. In DEX treated as well as in control ears the SGN density did not differ after 3 months therapy. Since we used an electrode insertion trauma model but Maini et al. investigated the SGN survival in normal hearing animals and in both studies different DEX concentrations were applied these may be explanations for the different results.

#### SGN soma diameter

Comparing the soma diameter of DEX treated and control ears no differences were observed in study I. However, in study II there were significantly larger SGN somata in the cochleae of DEX-treated ears compared to controls (10% DEX: p<0.05, 1% DEX: p<0.001), which applied to the whole cochlear length whereas in study III the soma diameter in DEX-treated cochleae were significantly lower than in controls (p<0.001). In the basal region of the same ears no soma diameter changes were observed. Since DEX is applied to the basal region only changes in soma diameter seem to have other causes than the DEX delivery. Moreover, we may speculate that DEX might have protected the basal region of the cochlea from these changes as it is believed that neuron shrinkage as well as increased diameter are signs for impaired vitality [42].

### Effect of DEX+ES on SGN

In study III local inner ear DEX-application was combined with chronic ES via CI. To our knowledge this experimental approach has not been done before. It is mimicking the clinical situation in CI patients where chronic ES is applied to the inner ear structures. ES was applied together with AP in one study group and increased the SGN density over the full cochlear length compared to the control group and DEX-only treated group. This neuroprotective effect of ES is known [25, 43–46] but its mechanisms are not exactly understood *in vivo* up to now. In *in vitro* studies using SGN cultures this trophic support of ES on SGN is explained by increased extracellular potassium levels, leading to prolonged periods of depolarization, promoting neuronal survival via activation of L-type voltage gated calcium channels [47, 48]. The resultant elevated intracellular calcium levels activate a number of down-stream pro-survival signaling pathways including cAMP protein kinase, and calcium/calmodulin-dependent kinases II and IV [49, 50].

In the present study the stimulation of the inner ear using charge balanced current pulses resulted in 3 cases of increased and 3 cases of decreased EABR-thresholds (AP+ES group). The simultaneous application of DEX and ES decreased the hearing threshold in 4 out of 5 animals and increased the SGN density of the total cochlea and of the basal region compared to control and DEX-only treated ones. Compared to sole ES treatment DEX+ES did not increase the SGN density of the full cochlear length. But when focusing on the basal turn, the region where DEX is delivered from the electrode array, a highly significant increase in SGN density is observed in DEX+ES treated ears compared to ES-only treated ones. This finding suggests that DEX and ES have a—direct or indirect—synergistic effect. An indirect effect may be the reduction of fibrosis since it was demonstrated that the degree of SGN loss is correlated with the degree of inflammation, and inflammatory responses have been described to promote loss of SGN [51, 52]. A mechanical blockage of the perilymphatic space with fibrous tissue may reduce circulation of the intra-cochlear fluids and consequently lead to accumulation of reactive oxygen species as has already been reported for endolymphatic hydrops [53]. Since it is reported that both ES and DEX [7, 8] can reduce intrascalar fibrosis we correlated fibrosis and SGN density of the different groups to investigate if ES or DEX indirectly triggered SGN survival via decreasing the formation of fibrous tissue in the scala tympani. Since in none of the treatment groups a correlation was observed we hypothesize that a direct effect, based on the simultaneous activation of the two different signaling pathways via the glucocorticoid receptor and voltage gated calcium channel activation, are responsible for the synergistic effect of DEX and ES on SGN survival.

The local trophic support of DEX in combination with ES leads to the assumption that DEX should be delivered over the full depth of cochlear implant insertion to allow for this synergistic effect over the full length. Incorporating DEX into the electrode array’s silicone may be the safest and most effective strategy for this attempt.

The hydrogel reservoir (study I) incorporated into a cochlear implant electrode array can be used as an alternative delivery strategy for drugs which cannot be mixed and released from the silicone of the electrode. Drugs and hydrogel can be manufactured to match for optimum release characteristics. For prolonged delivery a pump based delivery system (study III) can be used. But the pump approach has two major limitations if intended to be used for continuous application: 1) the reservoir has to be refilled periodically and 2) a pump offers a permanent, possible entrance for infectious agents to the inner ear [54]. Therefore the pump-delivery approach is up to now not applicable for clinical use.

### Discussion of methods

When comparing the median EABR thresholds on the day of implantation of the AP+ES and DEX+ES groups of study III we observed a significantly lower threshold of the AP+ES group (165μA) compared to the DEX+ES group (270μA). We have no explanation for this since animals were randomly assigned to one of the two experimental groups and both experimental groups were implanted in a random order. Additionally, the electrodes in both groups were identical. They were implanted by the same surgeon and the same equipment for surgeries and EABR measurements was used. Since the baseline EABR was measured directly after implantation we can exclude that the pump based delivery of either AP or DEX influenced the threshold.

In study I and II histology was performed on plastic embedded specimens. Here, the technique allows for leaving the implant in situ for grinding [7, 8]. In study III, where paraffin embedding was performed, the implants had to be removed before sectioning for histological evaluation of neuron density and fibrosis due to the fact that cutting is not possible with the electrode made of platinum in situ. Even though paraffin- or cryo-sectioning are widely used methods in inner ear histology [19, 20, 25, 55] including fibrosis evaluation [10, 39] it cannot be excluded that removal of the electrode array may lead to removal of fibrous tissue being attached to the electrode surface. It is possible that the electrode explantation causes false results due to manipulation of the amount of fibrosis. For analysis of pathological inner ear tissue growth we recommend to leave the implant *in situ* to avoid undesired tissue removal.

The DEX concentrations applied per hour locally into the cochlea within the studies presented here ranged from 25 pg (study III, pump), 0.66 ng and 2.04 ng (study II, silicone) to 0.35 μg (study I, reservoir) and lasted for four weeks (study I and III) or three months (study II).

Up to now we only knew of one clinical trial where DEX is applied locally into the inner ear (ClinicalTrials.gov Identifier: NCT02905305). This trial is in the recruitment phase and up to now no information about the DEX concentration released into the perilymph has been given. Therefore it is hardly possible to state which concentrations are necessary to be applied locally to evoke a biological response in the inner ear tissue of patients without causing negative side effects. Neither is it possible to calculate the concentration needed for local inner ear application on the basis of intratympanically applied DEX. DEX has been intensively investigated in clinical studies for its biological effects if applied intratympanically (e.g. [2, 3, 21, 56, 57]). However, it is not possible to calculate the intra-cochlear doses that were achieved in those studies based on the information given on applied concentrations, volumes and treatment regimen. To calculate the effective concentrations of DEX delivered locally into the cochlea pharmacokinetic studies on DEX diffusion through the round window membrane and other routes of inner ear entry like the stapes footplate have to be performed, the turnover rates of DEX in perilymph needs to be known. Additionally, the time course of the conversion of inactive DEX formulations like DEX 21-phosphate into the active molecule have to be investigated [58]. The concentrations chosen within the studies cover a wide range—from pg to μg—and resulted in the used animal model, the guinea pig, in reduced acoustic hearing thresholds (study I, previously published: [8]), impedances (study II, previously published: [7]) and in combination with ES in reduced electrical hearing thresholds (published here) but none of the concentrations did have a negative effect on the SGN.

## Conclusion

Based on our findings in three different guinea pig experiments that DEX has no effect on SGN density and soma diameter we consider DEX as safe within the relatively wide range of concentrations used in the present studies. Simultaneous application of DEX and ES has a synergistic neuroprotective effect which is limited to the region of DEX application, leading to the conclusion that DEX should be delivered over the full electrode length. Therefore our results suggest that DEX can be used to preserve SGN from degeneration, reduce fibrosis and preserve residual hearing in CI surgery.
